# Magnetic field induced orientational transitions in liquid crystals doped with carbon nanotubes

**DOI:** 10.3762/bjnano.8.280

**Published:** 2017-12-29

**Authors:** Danil A Petrov, Pavel K Skokov, Alexander N Zakhlevnykh

**Affiliations:** 1 Physics of Phase Transitions Department, Perm State University, Bukireva St. 15, 614990 Perm, Russia

**Keywords:** carbon nanotubes, liquid crystal, magnetic field, orientational transitions, soft coupling

## Abstract

We propose a continuum theory of orientational phase transitions induced by an external magnetic field in a suspension of carbon nanotubes in a nematic liquid crystal. It is shown that in a magnetic field a non-uniform and two different uniform phases are possible in the suspension. The uniform phases of the suspension differ by the type of orientational coupling of nanotubes with the liquid crystal matrix (the planar type when the nanotubes are oriented along the matrix director, and the homeotropic type when the nanotubes are perpendicular to the director). The possibility of a redistribution of the nanotube concentration (segregation effect) is shown. The fields of orientational transitions between uniform and non-uniform phases of the suspension are found analytically. It is shown that, when the nanotubes are weakly coupled to the matrix, the magnetic field induces reentrant transitions (uniform planar phase–non-uniform phase–uniform homeotropic phase–non-uniform phase). These transitions can be of first or of second order depending on the carbon nanotubes segregation intensity.

## Introduction

In recent years suspensions of anisometric particles in liquid crystals have become of great interest for researchers [1]. This is not only because liquid crystals (LCs) have found wide application in modern optoelectronic devices [2] but also because anisometric particles orient like a LC in a medium that is capable of spontaneous orientational ordering. Examples of such media are suspensions of ferromagnetic or ferroelectric particles, as well as of carbon nanotubes. In 1970, Brochard and de Gennes proposed to dope nematic liquid crystals (NLCs) with elongated ferromagnetic particles [3]. The magnetic susceptibility of such composite system (called ferronematic), turned out to be several orders of magnitude higher than that of the pure LC even for a low concentration of the dispersed phase (0.01 vol %). The new approach has opened the way for the creation of devices that operate on the basis of magnetic orientational transitions in the LC. To date, many theoretical and experimental studies on the properties of ferronematics have been published [4–5], which indicates the interest in this kind of composite materials.

Along with ferri- or ferromagnetic particles it is also possible to use carbon nanotubes (CNTs) in order to increase the magneto-orientational response of the LC matrix [6–7]. Due to the highly elongated shape (aspect ratios of 10^2^ to 10^3^) and anomalously high anisotropy of the diamagnetic susceptibility (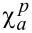 ≈ 10^−5^ to 10^−4^) [8–11], CNTs are very attractive for the creation of nanocomposites based on LCs with high magneto-orientational response. From experimental data [12–16], it is known that in the absence of external fields CNTs are oriented parallel to the director of the LC matrix, which corresponds to the planar type of coupling. However, homeotropic coupling is also possible [17]. Thus, for suspensions of CNTs based on LC with positive diamagnetic susceptibility anisotropies, one should expect decreasing of the threshold field of the magnetic Fréedericksz transition, which is confirmed by experiment [18–19], as well as a decrease in the electric field of the Fréedericksz transition [11,14,20–22]. Along with this, there are experimental studies devoted to the investigation of LC suspensions with CNTs functionalized by ferromagnetic particles [19,23–25], where an enhanced magneto-optical response is also observed in comparison with a pure LC.

The available theoretical approaches to the description of CNT suspensions in LCs are based on a generalization of the Landau–de Gennes theory [26–29] and mean-field theory [30–31]. In these papers, the phase state of the binary mixture of CNTs in LC as a function of the concentration of CNTs, the coupling energy of subsystems and temperature in the absence of external fields were studied.

In the present paper we propose a continuum theory of dilute CNT suspension in LCs that makes it possible to study orientational transitions induced by the magnetic field.

## Results and Discussion

### Basic equations

**Figure 1:**
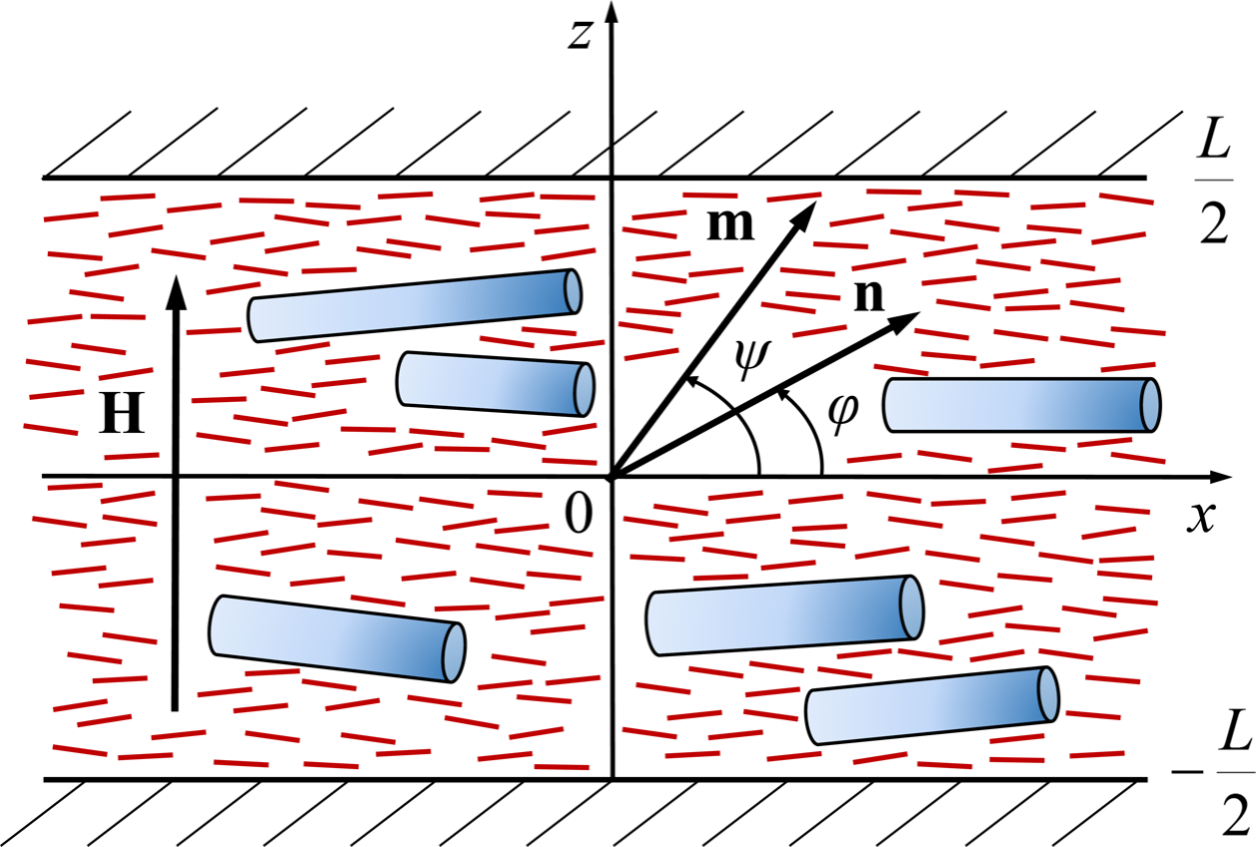
The planar layer of LC doped with CNTs in an external magnetic field, choice of the coordinate system.

We consider infinite plane layer of thickness *L* of a suspension of CNTs in NLC with planar texture and an absolutely rigid anchoring of NLC molecules with the boundaries. We set the origin of the coordinate system at the middle of the layer (see Figure 1). We use the unit vectors **n** and **m**, the so-called directors, to describe the preferential orientation of the LC molecules and CNTs, respectively. We assume soft and planar coupling of LC with the CNT surface, then in the absence of a magnetic field 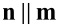. In the case of a positive anisotropy of the diamagnetic susceptibility of the LC χ*_a_* > 0, applying a magnetic field **H** = (0,0,*H*) will lead to the appearance of distortions in the orientational structure of the LC. This effect is known as the Fréedericksz transition [32]. CNTs are also oriented by the magnetic field, because they possess an anomalously strong diamagnetism and positive diamagnetic anisotropy [10,33–35], so even small concentrations of CNTs in suspension should lead to a decreasing of the Fréedericksz transition threshold in comparison with the pure NLC. These two mechanisms of the field influence on the suspension prove to be interdependent due to the orientational interaction of CNTs with the LC matrix.

In the framework of continuum theory, the equilibrium state of the suspension corresponds to the minimum of free energy

[1]
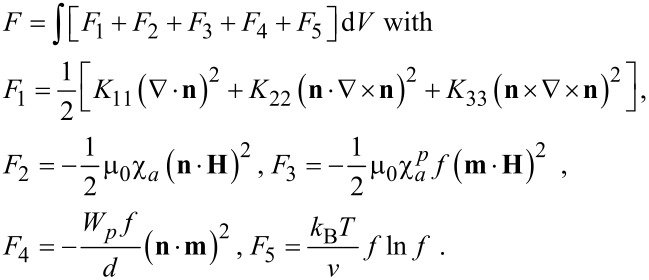


Here, *K*_11_, *K*_22_ and *K*_33_ are the Frank elastic moduli; χ*_a_* and 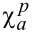 are the diamagnetic susceptibility anisotropies of LC and CNTs, respectively; μ_0_ is the permeability of vacuum; *f* is the volume fraction of CNTs in the suspension; *W_p_* is the surface density of the coupling energy between the LC molecules and the surface of the CNTs; *d* is the transverse diameter of a CNT; ν is the volume of a CNT; *k*_B_ is the Boltzmann constant; *T* is the temperature. We assume a low concentration of CNTs in LC, 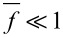 (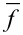 = *N*ν/*V*, N is the number of CNTs in the suspension, V is the suspension volume). This allows us to neglect the interaction between the CNTs.

The term *F*_1_ in Equation 1 is the free energy density of elastic deformations of the LC [36]. The contributions *F*_2_ and *F*_3_ take into account the interaction energies of the LC matrix and CNTs with the magnetic field. The term *F*_4_ describes the interaction of elongated impurity particles (in our case CNTs) with LC molecules [37]. In the absence of a field and for *W_p_* > 0, the minimum of *F*_4_ corresponds to 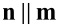, i.e., a planar coupling of the LC matrix and CNTs. The planar type of coupling was observed in most of the experimentally studied suspensions of CNTs in LCs [14,18,38]. The last term *F*_5_ is the contribution of the entropy of mixing of an ideal solution of CNTs in the LC matrix.

As it is known, the state of thermodynamic equilibrium corresponds to the minimum of the free energy (Equation 1), which is a functional of the two vectors **n** and **m**, and the scalar quantity *f*. Thus, the problem is reduced to deriving the equilibrium configurations of the directors **n**(**r**) and **m**(**r**), and the volume fraction *f*(**r**) of the CNTs.

In the considered geometry, the vectors **n** and **m** conveniently have the following form:

[2]
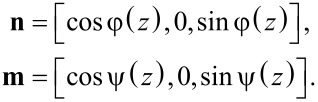


We choose the thickness *L* of the layer as the unit of length and the quantity 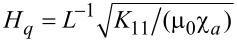 as the unit of the magnetic field strength, which corresponds to magnetic Fréedericksz transition field in pure NLC. We introduce the following dimensionless parameters [39]:

[3]
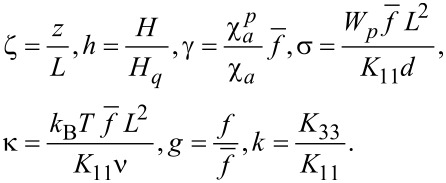


Here, ζ is the dimensionless coordinate and *h* is the dimensionless magnetic field strength. Due to the coupling between the directors of LC, **n**, and CNT, **m**, (term *F*_4_ in Equation 1), the suspension possesses two orientation mechanisms under the influence of a magnetic field. Both mechanisms are quadrupole in nature and are caused by the diamagnetism of the LC matrix and the CNTs. The parameter γ, which is determined by the balance of the terms *F*_3_ and *F*_2_ of the free energy (Equation 1), characterizes the relative contribution of the mechanisms of magnetic field influence on the orientational structure of the suspension. For 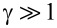, the appearance of orientational distortions of the director field are caused mainly by the diamagnetism of the CNTs, and for 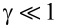, the distortions of orientational structure arise mainly from the diamagnetism of the LC matrix. The parameter σ characterizes the coupling energy of the LC and the impurity subsystem; *g* is the reduced volume fraction of CNTs in the suspension, and *k* is the ratio of Frank's constants of the LC.

The parameter κ is the square of the ratio between two characteristic lengths, i.e., layer thickness *L* and segregation length 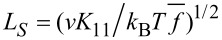 [3,40]. The characteristic size of the concentration redistribution region, *L_S_*, can be determined from the balance of the contributions *F*_1_ and *F*_5_ in the volume density of the free energy (Equation 1), which allows us to introduce the dimensionless segregation parameter 
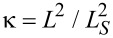
. For 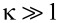, the segregation effect is negligible because the characteristic scale of the segregation region of CNTs considerably exceeds the thickness of the layer; for κ ≤ 1, the segregation effect becomes significant.

We take typical values for NLCs [36] to estimate dimensionless quantities: χ*_a_* ≈ 10^−6^ and *K*_33_ > *K*_11_ ≈ 10^−12^ N. For CNTs we can assume [7–11]: 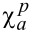 ≈ 10^−5^ to 10^−4^, *d* ≈ 10^−8^ m, CNT length *l* ≈ 10^−6^ m and ν ≈ 10^−22^ m^−3^. We also set *T* = 300 K and *L* = 20 μm. For different suspensions the coupling energy of CNTs with the LC matrix, *W_p_*, varies over a wide range from 10^−7^ N·m^−1^ [26] to 10^−3^ N·m^−1^ [24]. Assuming a volume fraction of CNTs 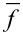 ≈ 10^−3^ [7], we obtain κ ≈ 1, γ ≈ 10^−2^ to 10^−1^, σ ≈ 1 to 10^3^ and *k* ≈ 1.

The dimensionless free energy takes the following form after the substitution of Equation 2 into Equation 1:

[4]
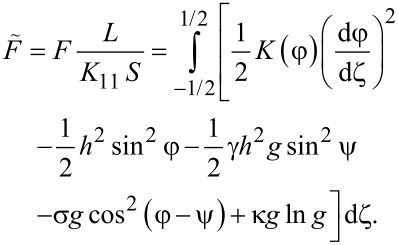


Here, *S* is the surface area of the planes confining the layer, and the notation *K*(φ) = cos^2^ φ + *k* sin^2^ φ is introduced.

Minimizing the functional (Equation 4) with respect to the functions φ(ζ), ψ(ζ) and *g*(ζ), we obtain a system of equations describing the orientational equilibrium of the suspension:

[5]
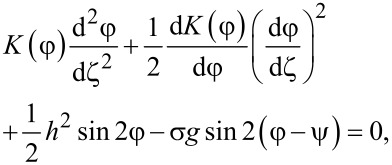


[6]
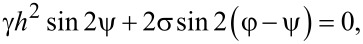


[7]
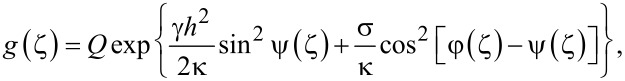


[8]
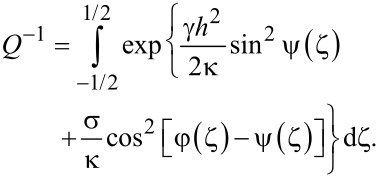


Here, the quantity *Q* can be derived from the condition of a constant number of CNTs in the suspension,

[9]
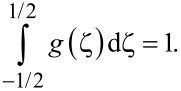


Together with the condition of a rigid planar coupling of the LC director to the boundaries of the layer,

[10]
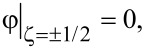


the system of Equations 5–8 allows us to find the equilibrium state of the CNT suspension in a magnetic field.

It follows from Equation 7 that in a suspension with non-uniform distribution of the directors **n**(**r**) and **m**(**r**), even a uniform magnetic field causes a redistribution of the CNT concentration *f*(**r**) in the layer, known as segregation effect [3] in the physics of ferronematics. This means that CNTs migrate to those regions of the layer where their magnetic energy in the field (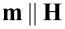) and the orientational energy in the LC matrix (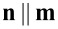) are minimal.

### Orientational phases of the suspension

**Figure 2:**
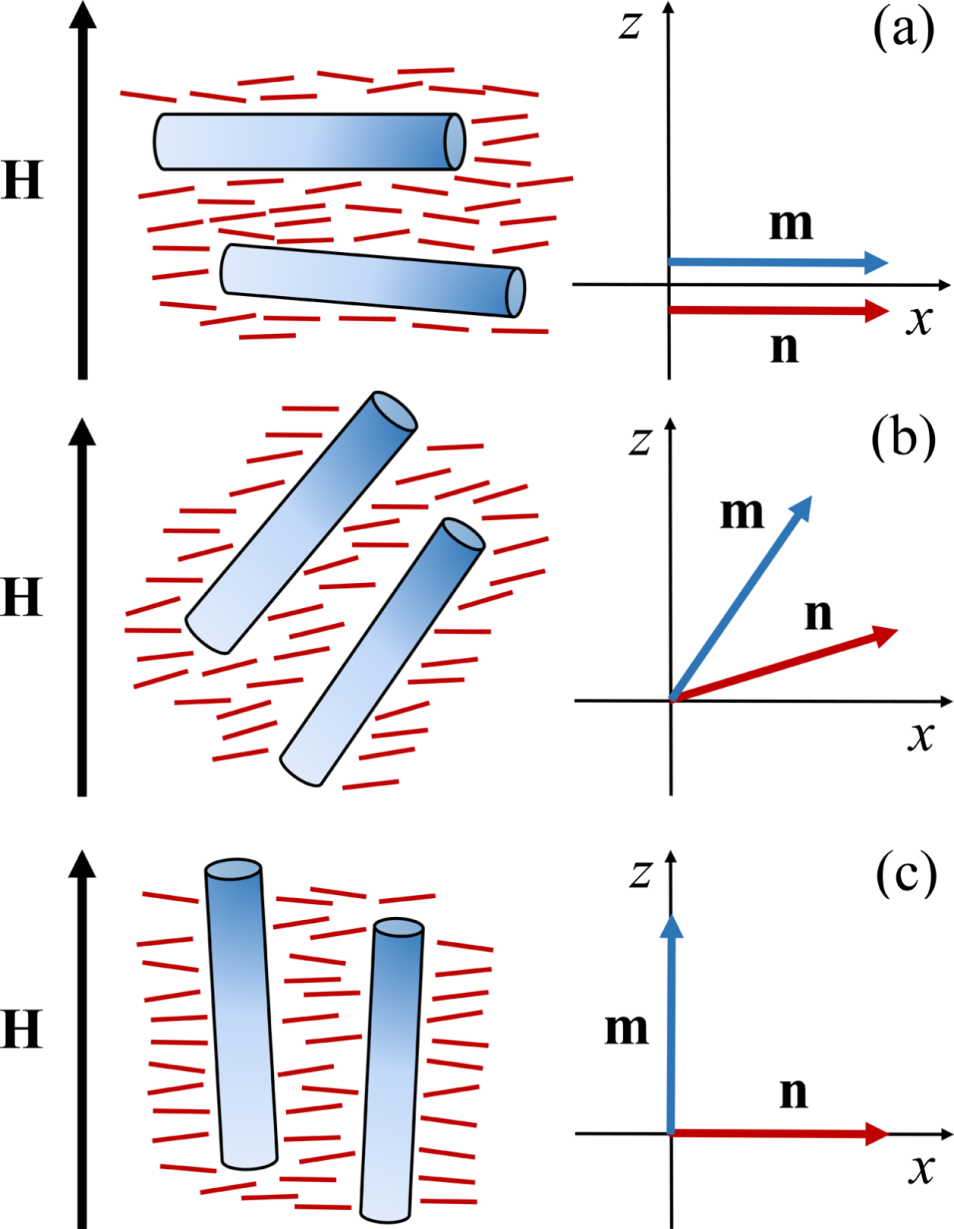
The structure of the orientational phases: (a) planar phase, (b) angular phase and (c) homeotropic phase.

The system of Equations 5–8 allows for uniform solutions [g(ζ) = 1 and the angles φ and ψ are independent of coordinates]. One of them [φ(ζ) = ψ(ζ) ≡ 0] corresponds to the initial state 
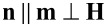
, in which the long CNTs axes are parallel to the director of the LC. We call this state the planar phase of the suspension, since it is characterized by the planar coupling (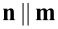) of CNTs to the LC matrix. In this state, the directors of the LC and CNTs are directed orthogonally to the external magnetic field and parallel to the boundaries of the layer. Another uniform solution [φ(ζ) = 0 and ψ(ζ) = π/2] corresponds to the homeotropic phase where the LC director is parallel to the layer boundaries and the CNT director is oriented along the field, 
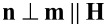
. This phase is characterized by the homeotropic coupling (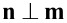) of CNTs to the LC matrix. The angular phase [41] corresponds to the non-uniform solution [φ = φ(ζ), ψ = ψ(ζ) and *g* = *g*(ζ)]. In this state the angle between the directors **n** and **m** is different from zero and π/2. Schematic representations of the planar, angular and homeotropic phases of the suspension are shown in Figure 2.

Under the influence of a magnetic field, the initial planar phase of the suspension (Figure 2a) becomes unstable. When the field reaches the threshold value *h_c_*, a transition to the angular phase occurs (Figure 2b) with distorted orientational structures of **n** and **m**. By analogy with pure LCs this transition can be called the Fréedericksz transition. The distortions of the orientational structure are small [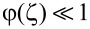, 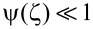] in the vicinity of *h_c_*, and the distribution of CNTs in the layer is almost homogeneous, *g*(ζ) ≈ 1. Therefore, the solution of the system of Equations 5–8 can be found analytically. In the lowest order we obtain φ(ζ) = φ*_m_*·cos(πζ) and ψ(ζ) = λ*_c_*φ(ζ). Here, the value of 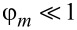 corresponds to the orientation angle of the LC director in the middle of the layer, and λ*_c_* is determined by the relation

[11]
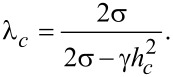


Non-zero solutions of φ(ζ) exist for h ≥ *h_c_*, where *h_c_* has the meaning of the Fréedericksz transition field from the planar to the angular phase and it is found from the following equation:

[12]
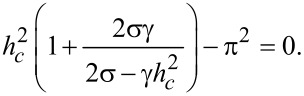


The thermodynamically stable solution of Equation 12 has the form

**Figure 3:**
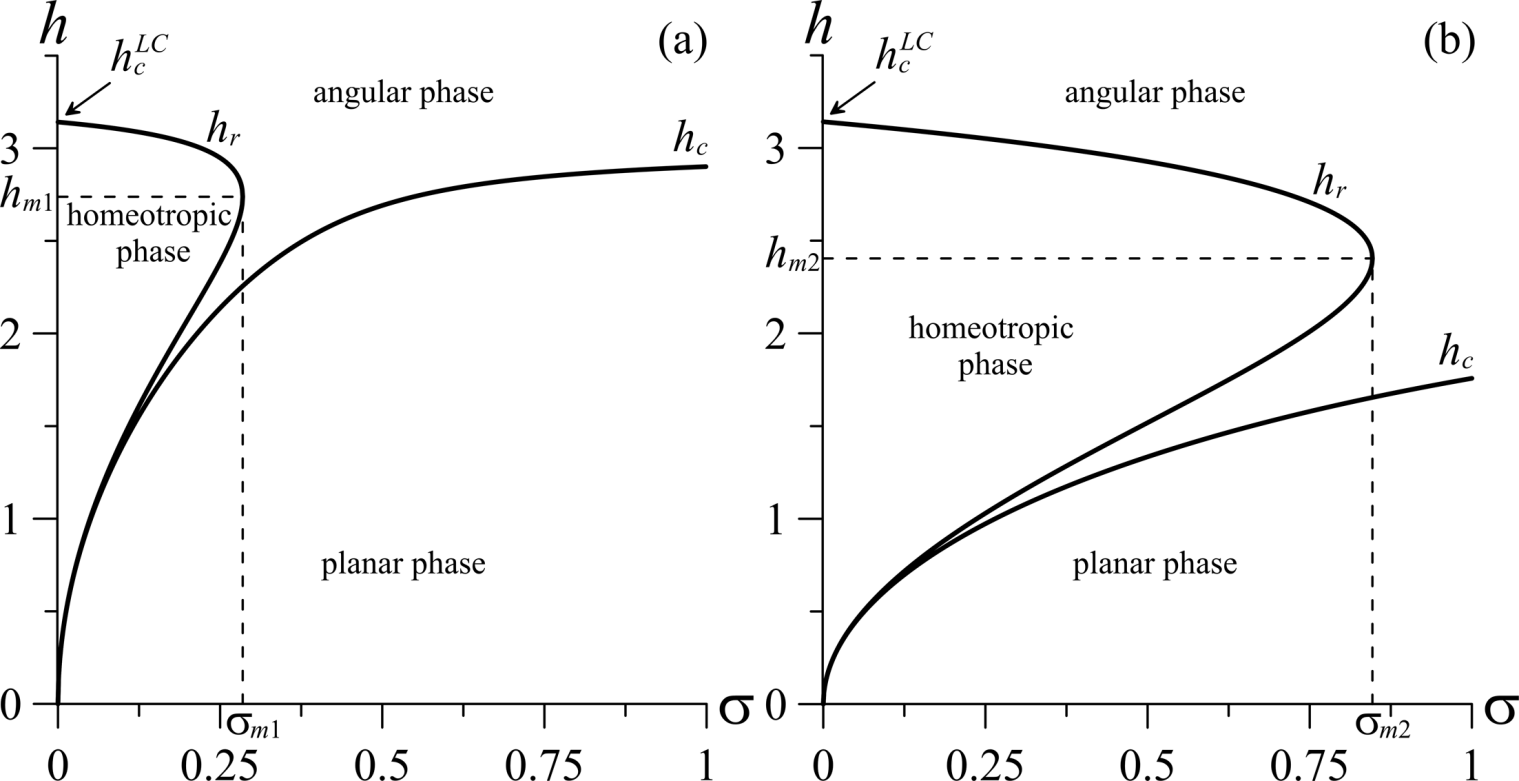
Diagram of the orientational state of the suspension for (a) γ = 0.1 [σ*_m_*_1_ = 0.285, *h_m_*_1_ = 2.738] and (b) γ = 0.5 [σ*_m_*_2_ = 0.847, *h_m_*_2_ = 2.404].

[13]
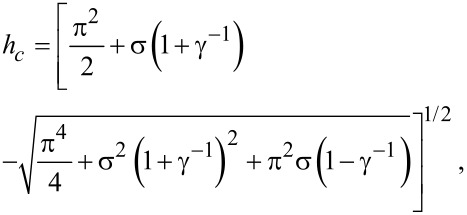


and it is shown in Figure 3.

In the case of strong coupling between CNTs and LC matrix (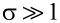), Equation 13 for the Fréedericksz transition field in the lowest order in the small parameter 1/σ gives

[14]
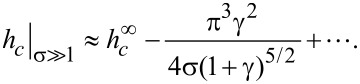


Here, the quantity 
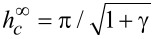
 corresponds to the field of the Fréedericksz transition in the case of absolutely rigid (σ→∞) coupling of LC and impurity subsystem, i.e., of the directors **n** and **m**. It follows from Equation 13 that with increasing parameter γ (i.e., as the volume fraction of CNTs or the anisotropy of their diamagnetic susceptibility increases), the Fréedericksz field decreases. Pure NLC with 
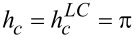
 corresponds to the value γ = 0.

When the CNTs is weakly coupled to the LC matrix in lowest order in small σ, from Equation 13 we obtain

[15]
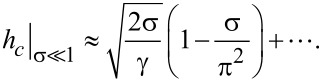


It follows from Equations 12–15 that for suspensions with planar coupling of the directors **n** and **m**, the Fréedericksz field is always smaller than for a pure NLC, which is confirmed by experiments [18–19,23].

We now determine the transition field between the non-uniform (angular) phase of the suspension and the uniform phase with the homeotropic coupling of CNTs with the LC matrix. In the homeotropic phase [φ(ζ) = 0 and ψ(ζ) = π/2] the LC director is parallel to the layer boundaries and the CNT director is oriented along the magnetic field (Figure 2c). In the vicinity of the transition field *h_r_* between the angular and homeotropic phases, the deviations of the director of the LC, **n**, from the *x*-axis and of the CNT director, **m**, from the magnetic field direction **H** are small. Therefore in the lowest order in small φ(ζ) and π/2 − ψ(ζ), we obtain the following equation for determining the transition field between the angular and the homeotropic phase:

[16]
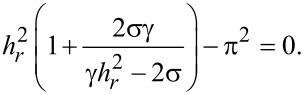


Equation 16 can be solved with respect to *h_r_* and we obtain

[17]
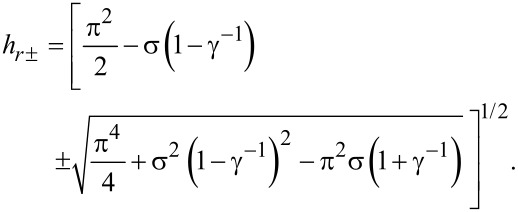


The solution *h_r_*_+_ describes the upper branch of the double-valued curve *h_r_*(σ) in Figure 3 and *h_r_*_−_ corresponds to the lower branch.

In the case of weak coupling of the CNT and LC directors (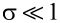) the expressions in Equation 17 can be represented in the form

[18]
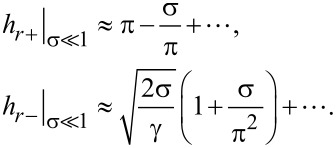


It is seen from Equation 18 that in fields 
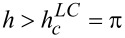
 the suspension can only be in the angular phase.

We note that the existence of the homeotropic phase is not possible for every value of the coupling energy of the LC matrix and CNTs, but only for σ ≤ σ*_m_*, where the threshold value of the coupling energy (Figure 3) is determined by the relation

[19]
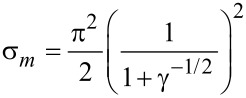


As can be seen from Equation 19, the region of existence of the homeotropic phase widens with increasing parameter γ. After substituting Equation 19 in Equation 16, we obtain the expression for the transition field *h_r_* corresponding to the coupling energy σ*_m_*:

[20]
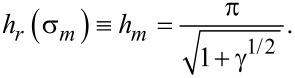


As can be seen from Equation 19, the upper bound of σ*_m_* is π^2^/2 (γ→∞). Therefore, for suspensions with the coupling energies σ > π^2^/2, the homeotropic phase cannot exist. For σ > π^2^/2, the coupling of CNTs with LC is almost absolutely rigid. In this case the description of the orientational structure of the suspension becomes possible with the help of one director **n** (**n** = **m**). It is seen from the expression for the free energy (Equation 1) that in this case the suspension behaves like a pure LC with an effective anisotropy of the diamagnetic susceptibility, 
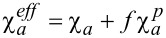
.

The diagram of the orientational state of the suspension, i.e., the threshold fields (Equation 13) and (Equation 16) for orientational transitions as functions of the coupling energy of CNTs with the LC matrix and for different values of γ is shown in Figure 3. The region bounded by the abscissa and curve *h_c_* corresponds to the planar phase in which the directors **n** and **m** are parallel to the boundaries of the layer (Figure 2a). The region bounded by the ordinate axis and the curve *h_r_* corresponds to the homeotropic phase in which the LC director is oriented along the layer boundaries and the CNT director is parallel to the magnetic field, 
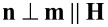
 (Figure 2c). Outside these regions is the angular phase with non-uniform distributions of **n** and **m** over the thickness of the layer (Figure 2b).

Figure 3 shows that if the coupling of CNTs with the LC matrix is weak (σ < σ*_m_*), there is a sequence of transitions with increasing field for a given value of the coupling energy: planar phase–angular phase–homeotropic phase–angular phase. That is, there are reentrant phenomena. Such reentrant transitions occur only for weak coupling of CNTs with the LC matrix. A magnetic field directed perpendicularly to the CNTs makes the initial alignment of tubes energetically unfavorable, so that they begin to rotate in the field direction minimizing the contribution *F*_3_ in Equation 1. The LC molecules also tend to rotate into the field direction minimizing the contribution *F*_2_. However, the orienting influence of the cell boundaries prevents a rotation of the LC director. Due to the orientational coupling between the LC and CNTs, the director rotation of the disperse phase (the term *F*_4_ in Equation 1) is transmitted to the LC matrix, and the first orientational transition from the initial planar phase to the non-uniform angular phase occurs at *h* = *h_c_*. The Fréedericksz transition threshold field, *h_c_*, appears to be smaller than that of the pure NLC. This result is in good agreement with experimental observations [18–19,23]. Due to induced gradients of the LC director and the increase in the energy of orientationally elastic deformations *F*_1_ in Equation 1, the forces of orientational elasticity tend to return the director to the initial planar state. Distortions of the orientational structure also cause the segregation of the CNTs, and the term *F*_5_ in Equation 1 is minimal when the impurity is uniformly distributed over the sample. Distortions of the LC director are induced in this case by the orientational coupling between **n** and **m** (contribution *F*_4_), i.e., the CNTs director entrains the LC director, and *F*_4_ tends to decrease the angle between **n** and **m**. For this reason, the appearance of large orientational deformations (i.e., large gradients of **n**) is energetically less favorable than a minimization of *F*_3_, leading to the occurrence of the uniform homeotropic phase with 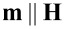. So that the next transition to the uniform homeotropic phase occurs with an increase of the magnetic field to *h* = *h_r_*_−_. For *h_r_*_−_ < h < *h_r_*_+_, the small energy loss (weak coupling) in the contribution *F*_4_ and *F*_2_ (the field is significantly lower than the Fréedericksz field of a pure LC) is compensated by the gains in the energy of orientational elastic deformations (*F*_1_ = 0), in the entropy contribution to the energy (*F*_5_ = min), and in the magnetic energy of CNTs (*F*_3_ = min). When the diamagnetism of the LC matrix (*F*_2_) begins to predominate, at *h* ≈ *h_r_*_+_, the next transition from the homeotropic phase to the non-uniform state (the angular phase) occurs. A similar diagram was described in [39] for ferronematic liquid crystals. In the case of strong coupling (σ > σ*_m_*), the initial planar phase undergoes a Fréedericksz transition to the non-uniform angular phase with increasing field. There is a “synchronous” rotation of the LC and CNT directors along the applied field, i.e., a monotonic increase in the deviations of the directors, since the energy loss in the contribution *F*_4_ is not small.

### Tricritical phenomena

We now determine the character of the orientational transitions. In the vicinity of the Fréedericksz field, *h_c_*, the deviations of the LC and CNT directors from the boundaries of the layer are small (small φ and ψ), so the free energy of the suspension (Equation 4) can be expanded in a power series over small φ(ζ) = φ*_m_*·cos(πζ) and ψ(ζ) = λ*_c_*φ(ζ). Here, the value 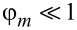 corresponds to the orientation angle of the LC director in the middle of the layer, and λ*_c_* is defined by Equation 11. After integration in the fourth order in φ*_m_*, the free energy takes the form of the Landau expansion

[21]
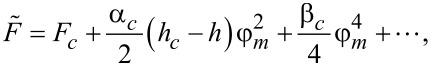


where

[22]
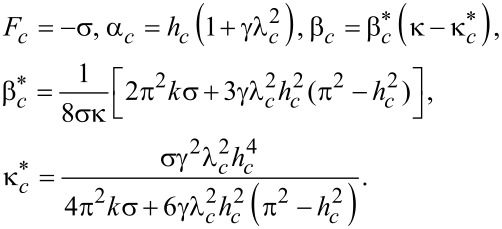


Here, *F_c_* is the free energy of the uniform planar phase.

The dependence of the LC director orientation angle in the middle of the layer, φ*_m_*, on the applied magnetic field in the vicinity of *h_c_* can be found by minimizing the expression in Equation 21 over φ*_m_*:

[23]
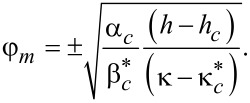


As noted above, for γ > 0 we always have *h_c_* < π, and therefore the coefficients of the expansion α*_c_* > 0, 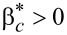 and 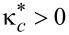. Then, as can be seen from Equation 23, the Fréedericksz transition between the planar and angular phases is a second-order transition (*h* ≥ *h_c_*) for 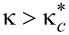. If 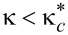, real solutions of Equation 23 exist only for *h* ≤ *h_c_*, and the Fréedericksz transition is a first-order transition. The quantity 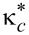 is the tricritical value of the segregation parameter below which the character of the Fréedericksz transition changes from second order to first order.

**Figure 4:**
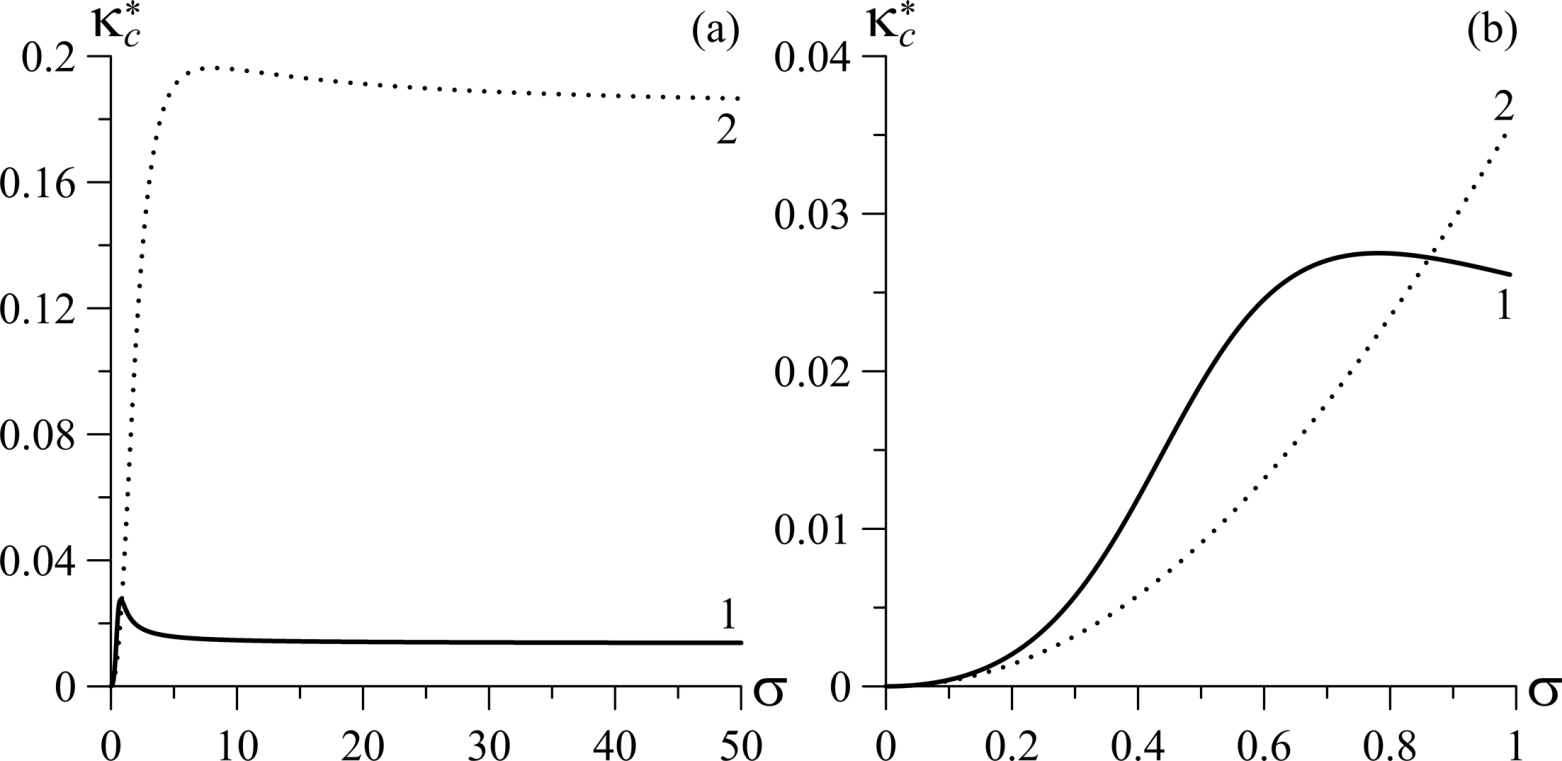
The tricritical segregation parameter 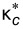 for the Fréedericksz transition as a function of the coupling energy of CNTs with the LC matrix, σ, for different values of the parameter γ for *k* = 1.5: curve 1 – γ = 0.1; curve 2 – γ = 0.5. (a) Large scale and (b) small scale.

The dependence of 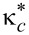 on the coupling energy of CNTs with the LC matrix for different values of γ is shown in Figure 4. For γ = 0.1 (curve 1) 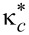 behaves non-monotonically with increasing coupling energy σ and exhibits a pronounced maximum, while for γ = 0.5 (curve 2) the maximum is poorly distinguishable. For σ > 10, 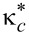 slightly varies for a given γ, and in the limiting case of rigid coupling of CNT and LC directors, it tends to the value of π^2^γ^2^/4*k*(1 + γ)^2^. It is seen from Figure 4a for σ > 10, 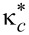 grows with increasing γ. For small coupling energies (Figure 4b), 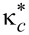 grows faster with increasing σ for γ = 0.1 than for γ = 0.5.

We now consider the transition from the angular to the homeotropic phase to which the field *h_r_*_−_ corresponds (see Figure 3). Near the transition point *h_r_*_−_, deviations of the LC director from the boundaries of the layer and of the CNT director from the direction of the magnetic field are small. Therefore, the free energy (Equation 4) can be represented in analogy with the previous case in the form of a Landau expansion:

[24]
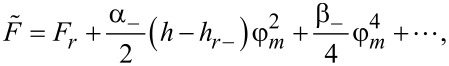


where

[25]
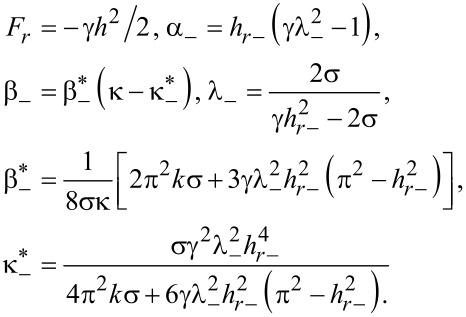


Here, *F_r_* corresponds to the free energy of the uniform homeotropic phase.

After minimizing Equation 24 with respect to φ*_m_*, we obtain the dependence of the LC director orientation angle in the middle of the layer on the magnetic field strength in the vicinity of *h_r_*_−_:

[26]
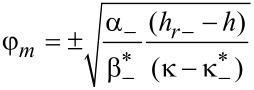


As can be seen from this formula, the considered transition is a second-order transition if real solutions of Equation 26 exist for fields h ≤ *h_r_*_−_. This is only possible for 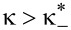, since the expansion coefficients α_−_ and 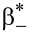 are positive for *h_r_* = *h_r_*_−_ < *h_m_*. For 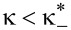, the transition from the angular phase to the homeotropic one is a first-order transition and the segregation parameter 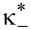 corresponds to the tricritical point.

Now we consider the possibility of changing the character of the orientational transition from the homeotropic to the angular phase, to which the field *h_r_*_+_ corresponds (see Figure 3). The Landau expansion of the free energy (Equation 4) in the vicinity of *h_r_*_+_ has the form

[27]
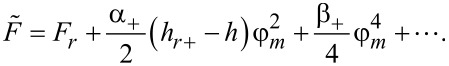


Here

[28]
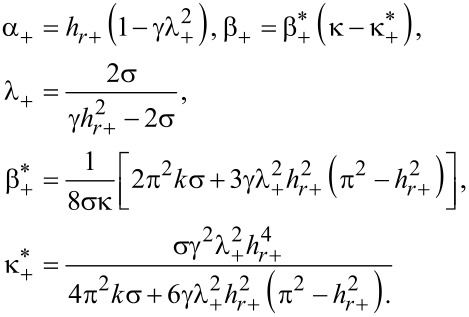


Minimizing Equation 25 with respect to φ*_m_* we obtain

[29]
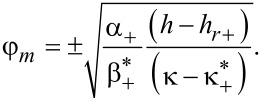


It can be seen that the transition of the suspension from the homeotropic to the angular phase with increasing magnetic field must be a second-order transition when real solutions of Equation 29 exist for *h* ≥ *h_r_*_+_ (see Figure 3), i.e., 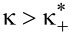 since α_+_ > 0 and 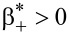. For 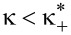 the transition from the homeotropic to the angular phase is a first-order transition. The quantity 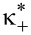 corresponds to the tricritical value of the segregation parameter κ.

**Figure 5:**
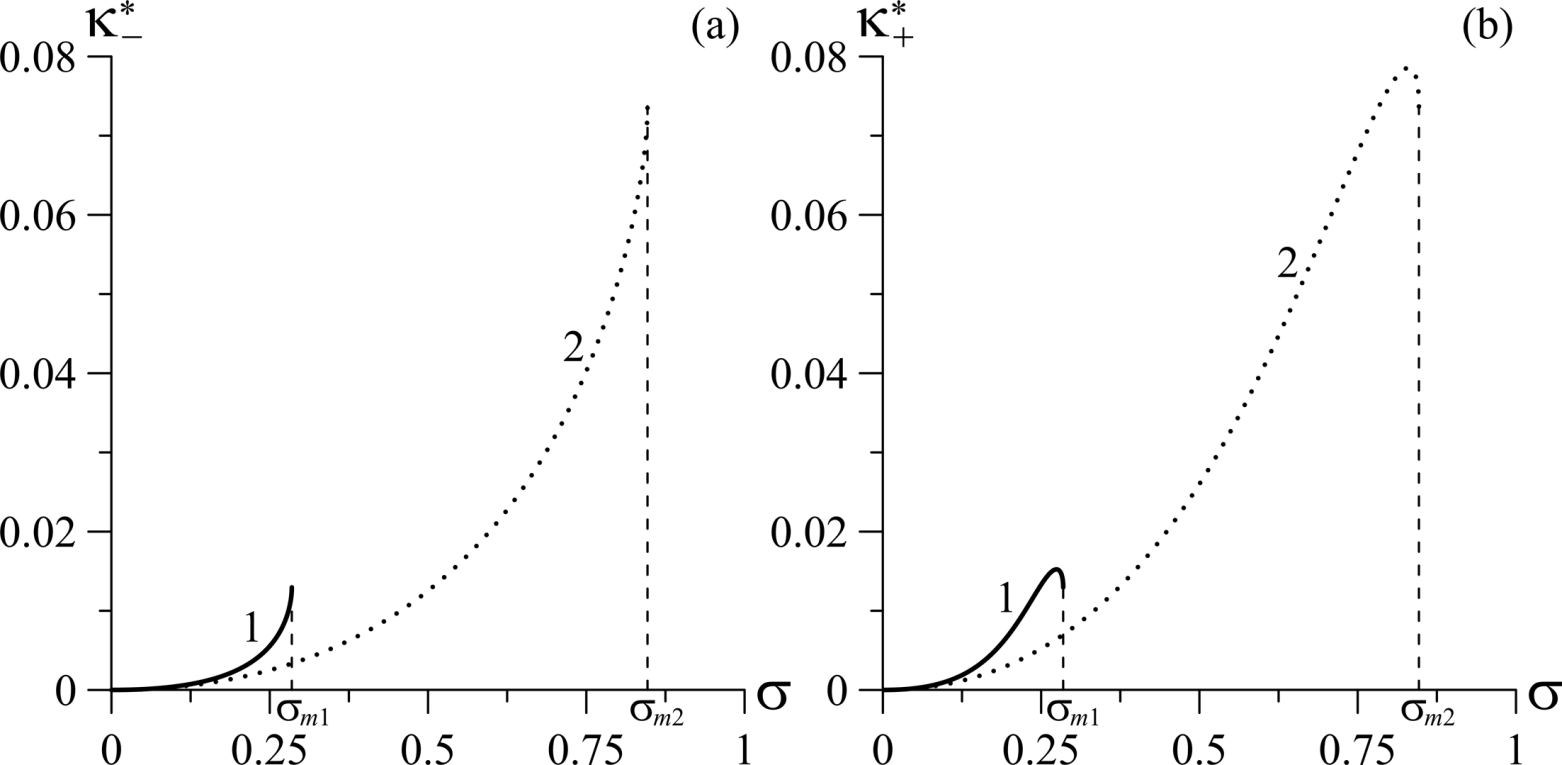
Dependence of the tricritical segregation parameters (a) 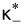 and (b) 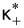 on the coupling energy of CNTs with the LC matrix, σ, for different values of the parameter γ for *k* = 1.5: curve 1 – γ = 0.1, σ*_m_*_1_ = 0.285; curve 2 – γ = 0.5, σ*_m_*_2_ = 0.847.

In Figure 5, the tricritical segregation parameters 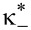 and 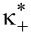 are given as functions of the coupling energy of CNTs with the LC matrix, σ, for different values of the parameter γ. 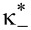 grows with increasing σ and γ (Figure 5a). The dependence of 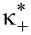 on σ and γ is analogous to that of 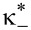 (Figure 5b) with the exception of the small region near σ ≈ σ*_m_* (right edge of the curves), where the 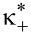 decreases with growing σ.

Equation 12 and Equation 16 allow us to determine only the threshold fields of the second-order transitions. To find the fields of the first-order equilibrium transitions, it is necessary to use the conditions for the equality of the free energy of the non-uniform state (Equation 4) and the free energy of the planar phase, *F_c_*, or of the homeotropic phase, *F_r_*, respectively.

## Conclusion

The orientational phase transitions in a suspension of CNTs in an NLC in a magnetic field perpendicular to the boundaries of the layer are studied. The possible phase states of the suspension in the magnetic field are established: a uniform phase with planar coupling of CNTs with the LC matrix, a non-uniform angular phase and a uniform phase with homeotropic coupling. Transitions between these phases occur when the magnetic field reaches certain threshold values. Analytical expressions that determine the fields of transitions between the uniform planar and non-uniform angular phases, as well as between the angular and the uniform homeotropic phases are found. It is shown that orientational transitions in the suspension occur at field strengths smaller than the Fréedericksz transition field of the pure NLC.

Furthermore, it is shown that when the CNTs are weakly coupled to the LC matrix reentrant transitions, planar phase – angular phase – homeotropic phase – angular phase, are possible under an increasing magnetic field. In the case of strong coupling the initial planar phase undergoes a Fréedericksz transition to the non-uniform angular phase with a growing magnetic field.

It is also shown that segregation takes place in the suspension of CNTs in the LC, i.e., under the influence of the magnetic field the CNTs are redistributed over the layer, so that the CNT concentration increases in those regions of the layer where the sum of their magnetic energy and the orientation energy of coupling to the LC matrix is minimum. It is found that, depending on the intensity of the segregation effects associated with the redistribution of CNTs over the layer thickness, all orientational transitions exhibit tricritical behavior, i.e., they can be transitions of both first- or second-order. The analytic expressions for the tricritical values of the segregation parameter are obtained.

The present paper is an extension to several studies of LC suspensions doped with dipole (ferromagnetic) particles [39,41–44]. Here we discuss the similarity and difference in the orientational behavior of dipole (ferromagnetic) and quadrupole (diamagnetic) particles in LC suspensions.

In the present paper we consider a physical system that, unlike ferronematics, does not have a dipole response to the applied magnetic field. We study LC suspensions with anisometric diamagnetic particles. Thus, the present theory describes the behavior of quadrupolar particles embedded in an LC. We analyze another possible way to enhance the anisotropy of diamagnetic susceptibility of LCs by doping them with diamagnetic CNTs.

In the magnetized ferronematics (in which the magnetic moments of the ferroparticles are aligned in one direction) with planar coupling of impurity particles to the LC matrix, the magnetic field induces non-threshold Fréedericksz transition for suspensions based on NLC with negative [42] or positive [39] anisotropy of the diamagnetic susceptibility. As we have shown above, in LC suspensions of CNTs this transition has threshold behavior. The next general feature of all considered systems is the possibility of existence of uniform homeotropic phases. In ferronematics with negative diamagnetic anisotropy of the LC matrix, the homeotropic phase remains stable with increasing field, while for positive diamagnetic anisotropy the transition from the homeotropic to the non-uniform angular phase occurs like for the CNT suspension. For all kinds of suspensions, the transitions between the angular and the homeotropic phases can be of first or of second order depending on the segregation intensity.

The so-called compensated ferronematics, which have equiprobable distributions of the particles parallel and antiparallel to the LC director in the absence of a field [43], show a magneto-orientational response which is similar to that of CNT suspensions in LCs. The compensated ferronematics exhibit a quadrupole response to the magnetic field applied perpendicularly to the initial alignment of the magnetic subsystems. They exhibit a threshold Fréedericksz transition from the initial compensated phase (planar phase) to the non-uniform angular phase, like the CNT suspensions. For weak coupling of magnetic particles and LC matrix the following sequence of reentrant transitions takes place: initial uniform compensated phase (planar phase) – non-uniform phase (angular phase) – uniform saturation phase (homeotropic phase) – non-uniform phase (planar phase). For strong coupling of ferroparticles with the LC matrix there is an analogous response of the LC director and magnetization to the external magnetic field, and the sequence of transitions is: uniform compensated phase (planar phase) – non-uniform phase. There are some very important differences between compensated ferronematics and the suspension of CNTs in the LC. For compensated ferronematics the Fréedericksz transition can be only a second-order transition, while for the CNT suspension this transition can be of first or of second order, depending on the segregation parameter. The other feature is that the transition fields for compensated ferronematics depend on the segregation parameter, while for magnetized ferronematics and for LC suspensions of CNTs there is no such dependence [39,42–43].

In [44] we studied the orientational response of a magnetized ferronematic liquid crystal to magnetic and electric fields. In contrast to the above-mentioned works, we considered the bistable coupling between the particles and LC matrix. It is shown that apart from magnetic impurity segregation, the first-order orientational transitions can be due to the bistable orientational coupling.

Another approach for studying the LC suspensions of the dipolar (ferromagnetic) particles was proposed in [45–46]. This approach is based on the mean-field theory, and allows for an investigation of the influence of temperature and magnetic field on a suspension, including the phase transition from the ordered phase into the nematic or paranematic state. We plan to propose such a theory for LCs doped with diamagnetic particles.
